# *cis*-4-[^18^F]fluoro-L-proline Molecular Imaging Experimental Liver Fibrosis

**DOI:** 10.3389/fmolb.2020.00090

**Published:** 2020-05-15

**Authors:** Qi Cao, Xin Lu, Babak Behnam Azad, Martin Pomper, Mark Smith, Jiang He, Liya Pi, Bin Ren, Zhekang Ying, Babak Saboury Sichani, Michael Morris, Vasken Dilsizian

**Affiliations:** ^1^The Department of Diagnostic Radiology and Nuclear Medicine, University of Maryland School of Medicine, Baltimore, MD, United States; ^2^Division of Nuclear Medicine and Molecular Imaging, The Johns Hopkins PET Center, Baltimore, MD, United States; ^3^The Russell H. Morgan Department of Radiology and Radiological Science, Johns Hopkins School of Medicine, Baltimore, MD, United States; ^4^Department of Radiology and Medical Imaging, University of Virginia, Charlottesville, VA, United States; ^5^The Department of Pediatrics in the College of Medicine, University of Florida, Gainesville, FL, United States; ^6^The Department of Surgery, University of Alabama at Birmingham School of Medicine, Birmingham, AL, United States; ^7^The Department of Medicine, University of Maryland School of Medicine, Baltimore, MD, United States

**Keywords:** molecular imaging, early stage alcoholic liver fibrosis, PET/CT, hepatic stellate cells, cis-4-[^18^F]fluoro-L-proline, steatohepatitis

## Abstract

**Introduction:** Early-stage liver fibrosis is potentially reversible, but difficult to diagnose. Clinical management would be enhanced by the development of a non-invasive imaging technique able to identify hepatic injury early, before end-stage fibrosis ensues. The analog of the amino acid proline, *cis*-4-[^18^F]fluoro-L-proline ([^18^F]fluoro-proline), which targets collagenogenesis in hepatic stellate cells (HSC), was used to detect fibrosis.

**Methods:** Acute steatohepatitis was induced in experimental animals by liquid ethanol diet for 8 weeks, intra-gastric binge feedings every 10th day along with lipopolysaccharide (LPS) injection. The control animals received control diet for 8 weeks and an equivalent volume of saline on the same schedule as the acute steatohepatitis model. First, *in vitro* cellular experiments were carried out to assess [^3^H]proline uptake by HSC, hepatocytes and Kupffer cells derived from rats with acute steatohepatitis (*n* = 14) and controls (*n* = 14). Next, *ex vivo* liver experiments were done to investigate unlabeled proline-mediated collagen synthesis and its associated proline transporter expression in acute steatohepatitis (*n* = 5) and controls (*n* = 5). Last, *in vivo* dynamic and static [^18^F]fluoro-proline micro-PET/CT imaging was performed in animal models of acute steatohepatitis (n = 7) and control (n = 7) mice.

**Results:** [^3^H]proline uptake was 5-fold higher in the HSCs of steatohepatitis rats than controls after incubation of up to 60 min. There was an excellent correlation between [^3^H]proline uptake and liver collagen expression (*r*-value > 0.90, *p* < 0.05). Subsequent liver tissue studies demonstrated 2–3-fold higher proline transporter expression in acute steatohepatitis animals than in controls, and proline-related collagen synthesis was blocked by this transporter inhibitor. *In vivo* micro-PET/CT studies with [^18^F]fluoro-proline showed 2–3-fold higher uptake in the livers of acute steatohepatitis mice than in controls. There was an excellent correlation between [^18^F]fluoro-proline uptake and liver collagen expression in the livers of acute steatohepatitis mice (*r*-value = 0.97, *p* < 0.001).

**Conclusion:** [^18^F]fluoro-proline localizes in the liver and correlates with collagenogenesis in acute steatohepatitis with a signal intensity that is sufficiently high to allow imaging with micro-PET/CT. Thus, [^18^F]fluoro-proline could serve as a PET imaging biomarker for detecting early-stage liver fibrosis.

## Introduction

The clinical course of alcoholic liver disease is critically dependent on the presence and extent of liver fibrosis. Therefore, it is extremely important to have a sensitive and reliable non-invasive imaging technique that can identify and monitor such hepatic fibrosis early, before end-stage liver fibrosis ensues. Alcohol consumption is a major cause of end-stage liver disease and the principal preventable cause of cirrhosis (Ismail and Pinzani, 2009). The spectrum of alcoholic liver disease encompasses alcoholic steatosis, alcoholic steatohepatitis, and alcoholic liver fibrosis. The degree of liver fibrosis is an effective prognostic factor useful in determining the degree of damage and assessing the potential of reversing the disease process (Friedman, 2000; Pratt and Kaplan, 2000; Friedman, 2008). Currently, there is no effective, non-invasive technique available to quantify the amount of fibrosis in the early stages of liver fibrosis. A biomarker is necessary for accurately diagnosing, staging, and monitoring disease progression and treatment response of early-stage liver fibrosis.

In the normal liver, hepatic stellate cells (HSC) are the primary site of vitamin A storage (as retinyl palmitate) and maintain serum vitamin A at physiologic levels (Knook et al., 1982; Moshage et al., 1990; Dufour et al., 2000a). However, alcohol consumption changes the gut bacteria microbiome, producing endotoxins such as lipopolysaccharride (LPS) and increases the permeability of the gut, resulting in elevated levels of portal LPS. LPS in the liver then activates Kupffer cells to produce inflammatory mediators such as tumor necrosis factor alpha, leading to liver inflammation and injury. LPS also stimulates the synthesis of fibrogenic mediators, such as transforming growth factor beta, leading to activation of quiescent HSC that transdifferentiate into myofibroblasts, producing collagen (Friedman, 2000; Ramadori et al., 2008). LPS has been used to induce acute liver injury in experimental acute alcoholic and non-alcoholic hepatitis in rat and mouse models (Batey et al., 1998; Lowe et al., 2017). Early-stage liver fibrosis, which cannot be clinically detected by current imaging techniques including computed tomography (CT) and magnetic resonance imaging (MRI), is a precursor to advanced cirrhosis. Both early-stage liver fibrosis and advanced cirrhosis involve HSC activation and collagen production (Friedman, 2000). Collagen contains approximately 15% proline. On a per gram basis, proline plus hydroxyproline are most abundant in collagen and play key roles for collagen stability. Consequently, [^3^H]proline has been used for *in vitro* and *ex vivo* assessment of collagen synthesis for more than four decades (Carneiro and Leblond, 1966).

In this study, we sought to determine whether [^18^F]fluoro-proline could be used to detect early-stage liver fibrosis using micro PET/CT imaging in experimental animals. The early stages of liver fibrogenesis do not reliably manifest as alterations in hepatic function due to the liver’s high compensatory reserve. For instance, serum markers of hepatocyte injury, such as alanine aminotransferase (ALT), aspartate amino transferase (AST), hyaluronic acid (HA) and alpha-2-macroglobulin (A2M) do not indicate the extent of fibrosis (Pratt and Kaplan, 2000). An ALT/AST ratio of 2:1 or greater has also been used to diagnose ALD, but none of these markers is useful in diagnosing early-stage liver fibrosis (Pratt and Kaplan, 2000). Similarly, the diagnosis and staging of liver fibrosis using a variety of serologic biomarkers, such as HA, A2M, matrix metalloproteinase-2, and type III procollagenic peptide have proven to be unreliable (Dufour et al., 2000a, b). Thus, the detection and quantification of liver fibrosis with [^18^F]fluoro-proline PET/CT molecular imaging early in the disease process may optimize pharmacologic intervention before end-stage liver fibrosis ensues.

## Materials and Methods

### Animals and Animal Feeding

All animals were housed in sterile cages and fed in a sterile hood at the University of Maryland School of Medicine and all procedures were approved by the Institutional Animal Care and Use Committee. Experimental imaging procedures and radiotracer operation in the project was approved by the Radiation Safety Operation Committee of University of Maryland School of Medicine. Normal and acute steatohepatitis and its control rats were used for *in vitro* and *ex vivo* experiments, and normal, acute steatohepatitis, and its control mice were used for *in vivo* experiments.

#### *In vitro* Experiments

In order to define a suitable [^3^H]proline radioactivity dose and incubation time for *in vitro* studies, [^3^H]proline uptake was measured in HSCs isolated from healthy 20–27 week-old female Sprague-Dawley (SD) rats (*n* = 7) (Charles River Laboratories, Wilmington, MA, United States), who had been fed Purina chow and water *ad libitum*. The source of [^3^H]proline was from PerkinElmer, Melville, NY, the position that the compound was tritiated and the molar activity at the point of use are as follows: L-[2,3-3H]-Proline (55–85 Ci/mmol). Rat livers yield significantly more HSCs per liver, when compared to mouse livers (Cao et al., 2002a,c,d). Next, we evaluated *in vitro* [^3^H]proline uptake by HSC, collagen type 1 levels in HSC culture medium, and mRNA expression of α1(1) procollagen type 1 by HSC. After HSC were treated with LPS, [^3^H]proline uptake by HSC, collagen type 1 levels in HSC culture medium, and mRNA expression of α1(1) procollagen type 1 were evaluated. Then we measured [^3^H]proline uptake by hepatocytes, Kupffer cells and HSC in acute steatohepatitis and control SD rats. Fourteen rats at 19 weeks of age were induced with acute steatohepatitis by feeding the animals liquid Lieber-DeCarli ethanol diet for 8 weeks (which provides 36% of calories as ethanol) with intra-gastric binge feedings of ethanol (2.5 g/kg body weight) every 10th day. Two days before the *in vitro* experiments, the acute steatohepatitis rats were intraperitoneally injected with LPS (Sigma, St Louis, MO, United States) at a dose of 10 μg/kg body (Cao et al., 2002a). The 14 control animals were fed a regular, isocaloric diet for 8 weeks and injected with an equivalent volume of saline on the same schedule as the acute steatohepatitis animals (Cao et al., 2002a, d).

#### *Ex vivo* Experiments

To use a mouse model of acute steatohepatitis for non-invasive imaging and to test specificity of [^3^H]proline uptake by HSC within the liver, five BALB/C mice were fed ethanol liquid diets for 8 weeks and received intragastric binge feedings of ethanol (2.5 g/kg body weight) on every 10th day and LPS injections (10 μg/kg body weight intravenously) 2 days before the *ex vivo* experiments. Five BALB/C mice were fed control diets for 8 weeks and an equivalent volume of saline was administered on the same schedule as the early stage liver fibrosis model. 100 mg wet weight liver slices were incubated in 3 mL buffer containing 0–0.8 mM proline, 0–2.0 mM proline transporter inhibitor benztropine, 25 mM Hepes, 50 U/mL penicillin, and 50 mg/mL streptomycin. Unless otherwise specified, 1.85 MBq [^3^H]proline was added to each incubation flask under an atmosphere of 95% O_2_ and 5% CO_2_ in a shaking bath, 30 cycles/minute, at 37°C for 90 min. [^3^H]proline in the liver slices was measured according to previously described methods (Li et al., 1992; van de Bovenkamp et al., 2005).

#### *In vivo* Experiments

To image liver collagenogenesis in the early-stage liver fibrosis model, a mouse model identical to the rat early-stage liver fibrosis model was used. The mouse model reduces the amount of imaging radiotracer doses and feeding costs but has the same early-stage liver fibrosis characteristics (Pennington et al., 1997; Cao et al., 1999; Hamarneh et al., 2017). Seven BALB/C mice underwent PET imaging of early stage liver fibrosis. Seven BALB/C mice underwent PET imaging as controls. The animal feeding was the same as used on the mice described in the *ex vivo* experiments.

### Liver Histopathology and Biochemistry Parameters of Alcoholic Liver Injury

To assess the overall liver architecture and the extent of liver fibrosis, histopathological evaluation of liver tissue was performed (Mazza, 2000; Wallace et al., 2002). Hematoxylin and eosin staining based on standard Elastica-van Gieson protocol were used to evaluate overall liver architecture and sections were stained with Masson’s trichrome stain to evaluate fibrosis (Cao et al., 1999; Ren et al., 2003; Hamarneh et al., 2017). The liver section slides were evaluated under a Nikon microscope (Y-THS, Japan) at 60× magnification, and photomicrographs were taken of stained liver sections. Each section was evaluated by pathologists through blinded assessment according to the Kleiner scoring system, with modifications as described in other studies [steatosis (0–3), lobular inflammation (0–3), ballooning (0–2), and fibrosis scores (0–3] (Pennington et al., 1997). Blood levels of ALT, AST, LPS, HA, and A2M were measured by commercial kits as described in our publications (Li et al., 1992; Cao et al., 2002a,b,c,d; van de Bovenkamp et al., 2005).

### [^3^H] Fluoro-proline Activity Measurements in HSC, Kupffer Cells, and Hepatocytes

HSC, Kupffer cells and hepatocytes were isolated from acute steatohepatitis and control rats according to our previously published methods (Cao et al., 2002b,c,d; van de Bovenkamp et al., 2005). Non-parenchymal cells from the liver by means of sequential *in situ* perfusion with collagenase and protease. HSCs were separated from other non-parenchymal cells over a discontinuous two-layer Nycodenz gradient (11.4 and 17%; Sigma Chemical, St. Louis, MO). Rat HSC at 0.5 × 10^5^ cells/mL, Kupffer cells at 1.0 × 10^6^ cells/mL, and hepatocytes at 0.5–1.0 × 10^5^ cells/mL were cultured for the experiments at 12 h, which is the earliest culture time post isolation used in order to mimic natural activation in the animal body; 19 mmol/L [^3^H]proline at 0–37 MBq/L was added to cultures under an atmosphere of 95% O_2_ and 5% CO_2_ at 37°C for up to 2 h. A liquid scintillation counter (PerkinElmer, Shelton, CT) was used to quantify [^3^H]proline according to previously described methods (Li et al., 1992). Briefly, after [^3^H]proline treatment, culture media was removed and cells were washed with cold Hank’s balanced salt solution buffer. The cells were lysed with 0.5 mL 0.5% Triton X-100. Cell lysates were scraped from the plate wells and collected in 1 mL tubes. After the samples were centrifuged at 12,000 rpm at 4°C for 10 min, 125 mL of suspended lysate was transferred into a 5 mL scintillation cocktail (Economical Biodegradable Counting Cocktail, RPI, Research Products International Corp., Mount Prospect, IL, United States) in a plastic scintillation tube and measured in a scintillation counter. Data were expressed as counts per minute (CPM)/mg protein. The viability of HSC, Kupffer cells and hepatocytes was ≥ 94 ± 1%, as determined by Trypan blue exclusion prior to the cells being seeded into culture plates.

### α1(1)Procollagen Gene Expression and Type I Collagen Concentration

Quantitative PCR (qPCR) was performed to assess mRNA expression of the α1(1) procollagen. Total RNA was isolated using QIAGEN RNeasy Mini Kit (QIAGEN, Valencia, CA, United States) from approximately a 100 mg sample of each animal liver and cultured HSC that were harvested after treatment as described in our published reports (Li et al., 1992; Cao et al., 2002c, d). Procollagen mRNA expression was calculated as relative fold change after normalization with 18S rRNA (Hs99999901; Applied Biosystems, Foster City, CA, United States). Based on previously described methods in the literature, enzyme-linked immunosorbent assay (ELISA) was used to quantify the concentration of type I collagen in HSC culture media and liver tissue specimens (Wallace et al., 2002; Ren et al., 2003; van de Bovenkamp et al., 2005). The range of detection used was 31.5–1000 ng/mL; data were expressed as mg/mL.

### Proline Transporter Gene and Protein Expression in Liver Tissue *ex vivo*

To study proline transporter expression in *ex vivo* liver tissue, RT-PCR was performed to assess mRNA expression of the proline transporter as described in other studies (Li et al., 1992; Cao et al., 2002c, d) Proline transporter protein expression was assessed using a polyclonal antibody against proline transporter (1:200) (Fisher, Waltham, MA, United States) (Cao et al., 2004; Niu et al., 2007).

### Radiotracer Synthesis of Cis-4-[^18^F]fluoro-L-proline

To detect HSC proline uptake *in vivo*, the PET radiotracer [^18^F]fluoro-proline was synthesized and used for micro PET/CT imaging experiments. [^18^F]fluoro-proline was selected as the proline radiotracer for *in vivo* experiments because its gamma emission (511 keV), high positron emission efficiency (97%) and its half-life (110 min) are ideal for PET/CT. Isotope [^18^F]fluoride was produced at the Johns Hopkins University PET center. Synthesis of [^18^F]fluoro-proline was performed at the Johns Hopkins University using previously described methods (Hamacher and Stocklin, 1995; Mazza, 2000; Wallace et al., 2002; Zimny et al., 2002). Briefly, in a typical non-carrier-added fluorination reaction, a solution containing 20 mg of Kryptofix 2.2.2 and 5 mg of K_2_CO_3_ in 1 mL of CH_3_CN:H_2_O (7:3) was eluted onto [^18^F]fluoride trapped on a QMA^TM^ cartridge. The resulting solution was dried under a stream of N_2_ at 95°C. This was followed by two cycles of azeotropic drying using 0.5 mL of anhydrous CH_3_CN. An 0.5 mL aliquot of CH_3_CN containing 10 mg of the (2S,4R) precursor was subsequently added to the dried [^18^F]fluoride and the mixture was heated at 110°C for 7 min in a Reacti-Therm heating block (Thermo Fisher Scientific, Walther, MA). The reaction mixture was then cooled and dried under N_2_ gas at 80°C. The radiolabeled intermediate was de-protected using 0.5 mL of 2 M trifluoromethanesulfonic acid at 145°C over 10min. After cooling of the reaction mixture to ambient temperatures, the acidic solution was neutralized using 0.5 mL of saturated KHCO_3_. [^18^F]fluoro-proline was purified using reversed-phase-high-performance liquid chromatography (RP-HPLC) (10 × 250mm Phenomenex Luna; 5 μm; NH2; 25% 0.01 M NaH_2_PO_4_ in CH_3_CN at 4.5 mL/min). The CH_3_CN in the collected fraction was removed using a cation exchange column (SCX; Biotage). [^18^F]fluoro-proline was eluted using 0.1 M Na_3_PO_4_ phosphate buffer. Decay corrected radiochemical yields were 40 ± 5% with radiochemical purities > 99%. Purified [^18^F]fluoro-proline was highly diastereomerically pure containing < 1% *trans*-4-[^18^F]fluoro-L-proline. Diastereomeric purity is a critical factor for *in vivo* imaging applications since the trans-stereoisomer will exhibit a vastly different biodistribution profile. While both isomers undergo renal excretion, the *in vivo* pharmacokinetic profile of trans-fluoro-proline more closely resembles that of an unnatural amino acid and is excreted without any incorporation into collagen (Wester et al., 1999). The radiochemical purity and molar activity of each batch of produced fluoro-proline tracer were determined by analytical RP-HPLC equipped with a radioactivity detector. HPLC quality analysis of pre-prepared purified [^18^F]fluoro-proline compared to a commercial sample of the chemical proline was performed prior to each PET/CT imaging study to determine radioisotope labeling purity. The radiopharmaceutical [^18^F]fluoro-proline was transported by a licensed company (Ecology Services Companies, Columbia, MD, United States) from Johns Hopkins University to the University of Maryland campus. Figure 4 shows representative HPLC chromatograms of the purified [^18^F]fluoro-proline (A) as well as a co-injection of the purified prepared [^18^F]fluoro-proline with commercial non-radiolabeled standard [^18^F]fluoro-proline to further confirm product identity (Figures 5B,C). The tracer was administered to animals intravenously as a phosphate buffer solution with a pH of 6.5–8 with minimal [^18^F]fluoro-proline molar activity of 1860 ± 15 MBq/μmol at the time of imaging experiments.

### [^18^F]fluoro-proline Positron Emission Tomography Study

PET/CT imaging was performed at the Core for Translational Research in Imaging at the University of Maryland School of Medicine. First, PET/CT dynamic imaging was used to assess [^18^F]fluoro-proline biodistribution in different organs at various time points in 5 normal mice at 14–16 weeks of age with body weights of 29 ± 2 g. The animals were injected with [^18^F]fluoro-proline (8 ± 1 MBq at a proline concentration of 19 mmol/L) via a tail vein catheter and subjected to immediate whole-body dynamic imaging from 0 to 60 min. Static [^18^F]fluoro-L-proline PET imaging was acquired during the 90–120 min period separately without additional tracer administration. To acquire liver HSC activation by static [^18^F]fluoro-proline imaging, the body weight of the acute steatohepatitis and control mice were measured and up to 11 MBq in 200 μl of [^18^F]fluoro-proline at the same proline concentration of 19 mmol/L was injected via a tail vein catheter. Static PET imaging acquisition for liver HSC activation was carried out over 30 min, 60 min after administration of [^18^F]fluoro-proline. After PET imaging, static CT images were acquired in the same animals. Multiplanar reformatted views of PET/CT images in the sagittal, coronal, and transaxial planes were performed to aid in anatomical co-localization and segmentation. The radiotracer biodistribution in 12 critical organs including the brain, lung, spleen, colon, stomach, pancreas, thigh, spine, heart, liver, kidney, and bladder was quantified by regions of interest (ROI) segmentation integrations. The data were analyzed at following time periods, 0–10, 10–20, 20–30, 30–60, and 90–120 min. PET data was calculated in megabecquerel per liter (MBq/L) and% injected dose (ID)/g of targeted organs or tissues to show the rate of tracer uptake.

To compare proline uptake of liver tissues in the setting of acute steatohepatitis and control mice, liver [^18^F]fluoro-proline activities were quantified as MBq/L,%ID/g, and SUV defined as percentage of the injected dose per gram of tissue per gram body weight (units:%ID.g-2) (Langen et al., 2005; Geisler et al., 2014). Data analyses of imaging were performed using the commercially available Inveon^TM^ Research Workplace PET image analysis software (Inveon PET, Siemens Medical Solutions, PA, United States).

### Statistics

Statistical analyses were performed using SigmaPlot 12 (Systat Software, Inc., San Jose, CA, United States). The continuous variables were summarized as mean ± standard error. To compare scalar variables between two groups, Student’s *t*-test was utilized (or the Mann–Whitney non- parametric test, as indicated). To compare a variable among three or more groups, the Analysis of Variance (ANOVA) or the Kruskal-Wallis non-parametric test was utilized. To evaluate the correlation between two scalar variables, Pearson correlation coefficients (r) were calculated. p ≤ 0.05 was considered statistically significant.

## Results

### *In vitro* Experiments to Determine the Optimal Dose and Optimal Time to Maximal Uptake of [^3^H]proline by HSCs

**FIGURE 1:**
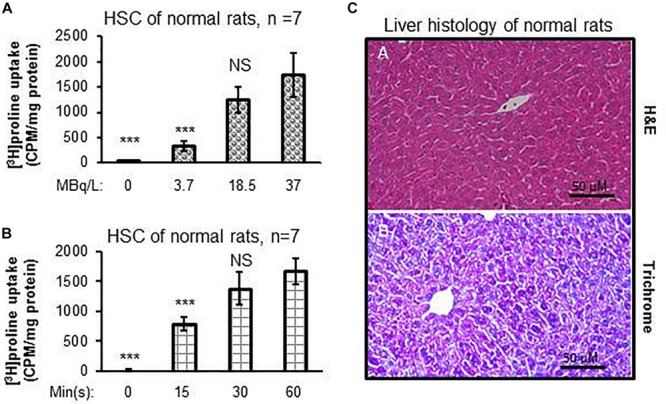
[^3^H]proline uptake by HSCs isolated from healthy rats is incubation time-dependent and liver histology is normal. **(A)** HSC [^3^H]proline uptake (*n* = 5), isolated from 7 healthy rats, was dose-dependent 30 min after incubation with different radioactivity doses of [^3^H]proline. Counts were acquired using a liquid scintillation counter and calculated into counts per minute with one milligram of HSC protein representing activation of collagen synthesis. ***p < 0.001 or NS vs. [^3^H]proline uptake by HSC of normal rats at the dose of 37 MBq/L **(A)**. **(B)** [^3^H]proline uptake is found to incubation time-dependent after HSCs (*n* = 5), isolated from 7 chow-fed rats, were incubated with 19 MBq/L of [^3^H]proline. ***p < 0.001 or NS vs. [^3^H]proline uptake by HSC at 60 min incubation. **(C)** representative photomicrographs of H&E staining **(A)** and reprehensive Trichrome staining **(B)** of liver tissue sections from healthy rats show no abnormal lipid, inflammatory, or collagen formation.

Dose-dependent differences were seen in proline uptake by healthy rat HSCs incubated with [^3^H]proline (Figure 1A). In addition, proline uptake by HSCs was greatest when HSCs were incubated with 19 MBq/L [^3^H]proline for 60 min as compared with shorter incubation times. However, there were no significant differences in proline uptake between the 30 and 60 min time points (Figure 1B). Taken together, we observed that proline uptake by HSCs isolated from livers of normal rats (Figure 1C) was dependent on both the cultured dose and time. The optimal [^3^H]proline dose was determined to be 19–37 MBq/L and the optimal time to maximize proline uptake by HSC was determined to be 30–60 min. This optimum dose and time for proline uptake by HSC was used for subsequent experiments.

### HSC Proline Uptake Significantly Correlates With α1(1)procollagen mRNA Expression and Type 1 Collagen Concentration in Culture Media

**FIGURE 2:**
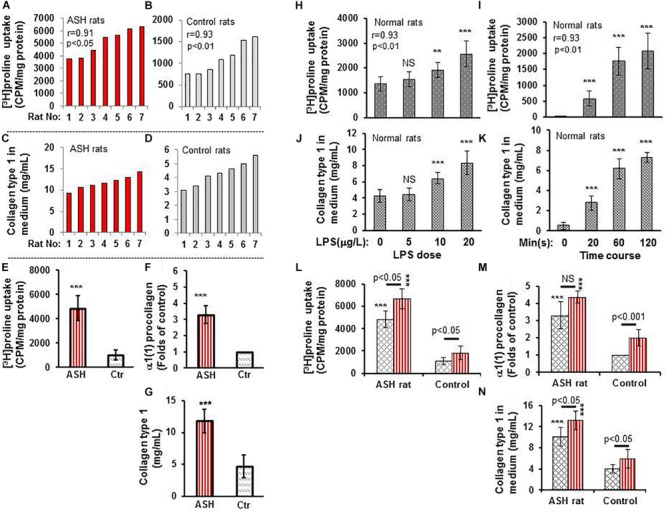
[^3^H]proline uptake and collagen type 1 expression by hepatic stellate cells (HSCs) isolated from rats with acute steatohepatitis vs. controls. **(A)** [^3^H]proline uptake by HSC isolated from 7 acute steatohepatitis rats; each red bar represents one acute steatohepatitis rat. **(B)** [^3^H]proline uptake by HSC isolated from 7 control rats; each gray bar indicates one control rat. Liquid scintillation radioactive counts were transformed into counts per minute in one-milligram HSC protein to represent activation of collagen synthesis. **(C)** collagen type 1 concentrations in culture media from HSCs of 7 individual acute steatohepatitis rats (red bars) using ELISA in the same order as in **(A)**. **(D)** collagen type 1 concentration in HSC culture media from 7 individual control rats (gray bars) using ELISA with the same animal order as **(B)**. Correlation *r*-values between [^3^H]proline uptake and collagen type 1 in acute steatohepatitis (*r*-value = 0.91) and controls (*r*-value = 0.93) were derived from the data in **(A–D)**. **(E–G)** [^3^H]proline uptake using a liquid scintillation counter, α1(1) procollagen mRNA using RT-PCR and collagen type 1 protein using ELISA looking at HSCs from acute steatohepatitis mice (red straight line bar, *n* = 7) and controls (gray slanted square bar, *n* = 7); ***p < 0.001 vs. [^3^H]proline uptake by HSC isolated from the controls at baseline 0. **(H,I)**, [^3^H]proline uptake by HSCs isolated from normal rats and treated with different doses (0–20 mg/L, *n* = 5) of LPS for 60 min and at different time points HSC incubated with 10 mg/L LPS for 0–120 min. NS, no significance, **P < 0.01 and ***p < 0.001 compared with HSCs not receiving [^3^H]proline (baseline 0 μg/L). **(J,K)** Collagen type 1 concentrations in control rat HSC culture media that were treated with different doses (0–20 mg/L, *n* = 5) of LPS and different time points (0–120 min). NS, no significance vs. control with no LPS added (0 μg/mL), **P < 0.01 and ***p < 0.001 vs. the values corresponding control at point of 0. Correlation *r*-values between [3H]proline uptake and collagen type 1 in relation to LPS dose (*r*-value = 0.93) and time course (*r*-value = 0.93) were derived from the data in **(H–K)**. **(L–N)**, [^3^H]proline uptake, α1(1) procollagen gene expression, and collagen type 1 concentration in HSCs isolated from acute steatohepatitis rats and treated and incubated with/without LPS, respectively; Red straight line bars represent HSC with LPS treatment (*n* = 5). Gray slanted square bars represent HSC with no LPS treatment (*n* = 5). ***p < 0.001 HSCs with LPS treatment (red straight bar) or no LPS treatment (gray slanted square bar) isolated from ASH animal livers vs. the corresponding controls.

*In vitro* experiments of cultured HSC isolated from the acute steatohepatitis rat group demonstrated significantly higher [^3^H]proline uptake than control rats (*p* < 0.001) (Figures 2A,B,E). Similarly, there was significantly higher type 1 collagen concentration in culture media (*p* < 0.001) (Figures 2C,D,G) and α1(1)procollagen mRNA expression (*p* < 0.001) (Figure 2F) in cultured HSC isolated from the acute steatohepatitis rat group compared to controls, consistent with HSC activation.

When the liver HSC stimulator LPS was added to cultured HSCs, increased [^3^H]proline uptake and collagen accumulation was present in a dose-dependent manner. The greatest [^3^H]proline uptake and greatest collagen accumulation was seen in HSC cultured with 20 mg/mL LPS for 60 min when compared with non-LPS-treated HSC (*p* < 0.001) (Figures 2H,J). There was a strong correlation between [^3^H]proline uptake and collagen accumulation (r = 0.93). [^3^H]proline uptake and collagen accumulation was the greatest in culture media after 120 min in 10 mg/mL LPS-treated HSC vs. non-LPS-treated HSC (*p* < 0.001) (Figures 2I,K). There was no significant difference in LPS treatment between 60 and 120 min. Therefore, the optimal dose and time for LPS treatment was 10 mg/mL LPS and 60 min, respectively. This dose and time were used for subsequent *in vitro* studies, which showed greater [^3^H]proline uptake, type 1 collagen gene expression, and collagen synthesis in the LPS-treated group compared to non-LPS-treated groups (Figures 2L–N).

### Histopathologic Analysis and [^3^H]proline Uptake by Hepatocytes, Kupffer Cells and HSC

Histopathological analysis of livers from the acute steatohepatitis rats confirmed the presence of steatosis and inflammation which was not present in control livers. Only minimal fibrotic collagen formation was seen in the acute steatohepatitis rat livers that were stained with H&E (Figures 2A,C), and fibrosis was confirmed in sections stained with Masson’s trichrome blue (Figures 2B,D). In contrast, no significant fibrotic collagen deposits were seen in control liver sections (Figures 2A,B).

**FIGURE 3:**
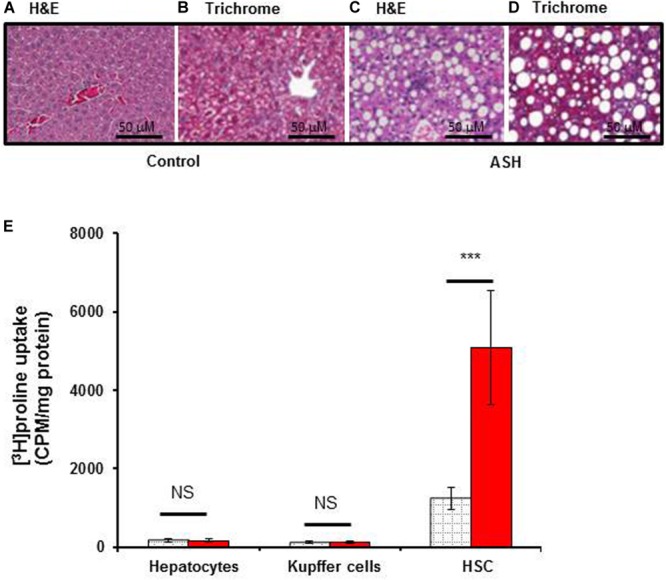
[^3^H]proline uptake by hepatocytes, Kupffer cells, and HSC isolated from rat with acute steatohepatitis vs. control rats, with liver histology. Photomicrographs of histologic liver sections from rats with steatohepatitis (ASH) vs. control rats. **(A,C)** H&E stained liver sections from ASH **(C)** and control **(A)** rats, 60× magnification. **(B,D)** Trichrome staining of liver sections from ASH **(D)** and control **(B)** rats showing increased collagen staining in the ASH liver section compared with the control section, 60× magnification. **(E)** [^3^H]proline uptake by hepatic stellate cells (HSC) was significantly higher in the steatohepatitis rats (*n* = 7) compared with the control rats (*n* = 7). ***p < 0.001 representing [^3^H]proline uptake by HSC between these two groups. Gray square bar, control rats. Red bar, acute steatohepatitis model rats.

To assess the proline uptake capacity in major populations of liver cells, we isolated HSC, Kupffer cells and hepatocytes from the livers of acute steatohepatitis rats and controls. As shown in Figure 3E, [^3^H]proline was preferentially taken up by HSC compared to hepatocytes or Kupffer cells. HSC isolated from the acute steatohepatitis rat livers (red bar) demonstrated significantly higher [^3^H]proline uptake compared to controls (gray bar, *p* < 0.001). In contrast, there was no significant difference in [^3^H]proline uptake by either hepatocytes or Kupffer cells isolated from the livers of acute steatohepatitis rats and control rats.

### An Acute Steatohepatitis Mouse Model for *in vivo* Imaging Studies and *ex vivo* Analysis of Proline Uptake Specificity by Collagen-Producing HSC

**FIGURE 4:**
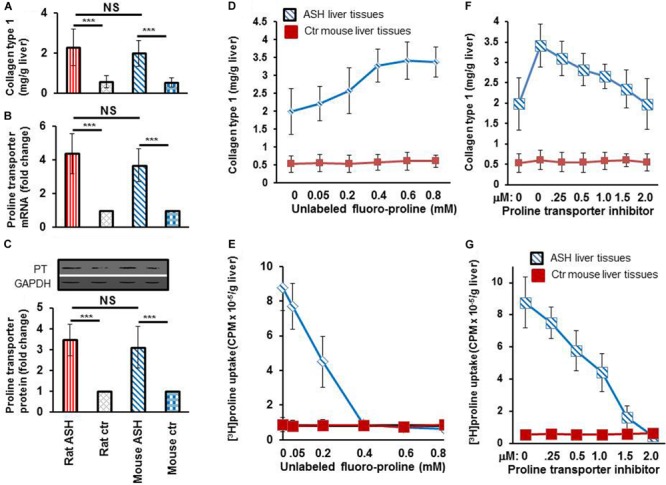
Collagen and proline transporter mRNA and protein expression in the livers of rats and mice with acute steatohepatitis and the assessment of proline transporter specificity of [^3^H]proline uptake by using unlabeled fluoro-proline and its transporter inhibitor. **(A)** Collagen levels in livers of rat (*n* = 7) and mouse (*n* = 5) with acute steatohepatitis (ASH) and controls. **(B)** Proline transporter mRNA expression at gene transcription level, and **(C)** proline transporter protein levels in livers of rats and mice with ASH. Investigate specificity of [^3^H]proline uptake in *ex vivo* liver tissue was investigated. **(D)** Liver collagen synthesis corresponding to different concentrations of unlabeled fluoro-proline in ASH mice (blue line) compared with control mice (red line), *ex vivo*. **(E)** Competitive transportation inhibition of liver [^3^H]proline uptake when unlabeled fluoro-proline (0–0.8 mM) and 1.85 MBq [^3^H]proline were incubated together with the liver tissues of mice with ASH (blue line, *n* = 5) compared with those of control mice (red line, *n* = 5), *ex vivo*. **(F,G)** Inhibition of unlabeled fluoro-proline-mediated liver collagen synthesis by the proline transporter inhibitor benztropine (0–2.0mM) in ASH mice (blue line, *n* = 5) compared with control mice (red line, *n* = 5), *ex vivo*. PT, proline transporter, ASH, acute steatohepatitis, Ctr, control. ***p < 0.001 vs. corresponding controls. NS, no significant difference between rats and mice with ASH.

After completing the *in vitro* studies in rats with acute steatohepatitis, we used an acute steatohepatitis mouse model for the *in vivo* imaging studies. Greater collagen accumulation was seen in the livers of rats with steatohepatitis compared with those of control rats (2.3 ± 0.9 vs. 0.6 ± 0.3 mg/g liver, *p* < 0.001), which was similarly seen in the mice with steatohepatitis vs. control mice (2.0 ± 0.6 vs. 0.5 ± 0.2 mg/g liver, *p* < 0.001) (Figure 4A). However, no significant difference in collagen synthesis was observed between the rat and mouse acute steatohepatitis models. In addition, proline transporter gene (Figure 4B) and protein expression (Figure 4C) was increased 3–4-fold in the livers of both the rat and mouse steatohepatitis models compared with corresponding controls. Again no significant difference in proline transporter gene and protein expression was identified between rat and mouse models.

To investigate the specificity of [^3^H]proline uptake by collagen-producing HSC, we first performed *ex vivo* experiments on liver tissues from acute steatohepatitis mice and control mice, in which we incubated the liver tissues with unlabeled proline. The data showed a gradual increase in collagen synthesis from 2.0 ± 0.6 to 3.3 ± 0.4 mg/g liver in the presence of 0–0.4 mM unlabeled proline. Collagen synthesis became consistently increased (3.3 ± 0.4, 3.4 ± 0.5, and 3.4 ± 0.4 mg/g liver) when 0.4–0.8 mM unlabeled fluoro-proline was used (Figure 4D). In contrast, in the presence of unlabeled proline, [^3^H]proline activity decreased to the levels of corresponding control liver tissues (0.9 CPM × 10^–5^/g liver) at 0.4 mM or greater concentration of unlabeled fluoro-proline from the peak activity of 8.8 CPM × 10^–5^/g liver at the baseline 0 mM unlabeled fluoro-proline (Figure 4E), demonstrating competitive inhibition of fluoro-proline transportation via its cellular membrane transporters.

Our results showed greatest collagen synthesis at an unlabeled fluoro-proline concentration of 0.6 mM (second square) compared to the baseline of collagen levels at the baseline 0 mM unlabeled fluoro-proline (2.0 ± 0.6 mg/g liver) (first square, Figure 4F). Fifty percent of proline transporter activity was blocked at a concentration of 0.5 mM benztropine and proline transporter activity was completely blocked at a concentration of 2.0 mM benztropine (Figure 4G).

### [^18^F]fluoro-proline Biodistribution Assessment Using Dynamic and Static Micro-PET/CT Imaging

**FIGURE 5:**
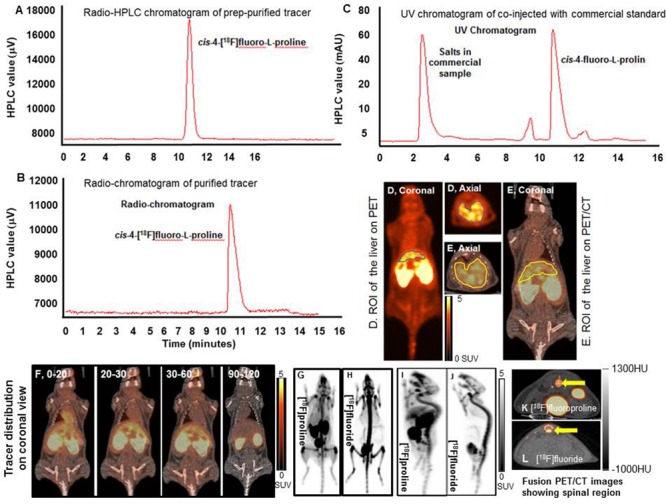
Dynamic and static [^18^F]fluoro-proline PET/CT and [^18^F]fluoride PET imaging. **(A–C)** histograms show HPLC analysis of prep-purified tracer, and final purified tracer formulation, co-injected with commercial standard non-radiolabeled fluoro-proline. **(D)** A region of interest (ROI) of the liver on PET image. **(E)** ROI of the liver on whole body fused PET/CT image. **(F)** [^18^F]fluoro-proline distribution time course in different organs using dynamic and static PET/CT imaging of normal mice. The representative normal mouse body weight was 31 g and the injected dose was 9 MBq of [^18^F]fluoro-proline. **(G,H)** Comparison between [^18^F]fluoro-proline and [^18^F]fluoride distributions in the livers and other organs of healthy mice (MIP views are representative of the same normal mouse). The representative mouse body weight was 31 g and the injected dose was 9 MBq of [^18^F]fluoro-proline and 10 MBq of [^18^F]fluoride at the time of imaging experiments. **(I,J)** A comparison between the distributions of [^18^F]fluoro-proline **(I)** and [^18^F]fluoride **(J)** in the spinal region (yellow arrow) of the same normal mouse on two axial views. MIP, maximum intensity projection.

HPLC demonstrated high ^18^F incorporation and high purity of synthesized [^18^F]fluoro-proline (Figures 5A–C). The biodistribution of [^18^F]fluoro-proline in several organs was evaluated in a normal mouse by drawing a region of interest (ROI) containing each organ/tissue, and then megabecquerel per liter (MBq/L) activity was measured within each ROI. Figures 5D,E depicts an example of how ROI was drawn for the liver on a whole body PET/CT from a normal mouse. There is marked [^18^F]fluoro-proline activity in the liver from the time of the initial imaging to 30 min post imaging, with visualization of subsequent clearance of [^18^F]fluoro-proline from the liver over the next 30 min (30–60 min time interval) with detectable intrahepatic radiotracer activity for up to 120 min after injection. The lack of tracer activity in the gallbladder during the imaging period is in agreement with the non-biliary clearance of [^18^F]fuoro-proline.

A complete summary of radiotracer uptake (MBq/L) in the evaluated organs from normal mice is presented in Table 1. The cardiac blood pool had the highest activity at the beginning of the imaging period, which was essentially washed out from the left ventricle in 30 min. The highest overall activity was present in the kidneys (between 10 and 30 min) and the urinary bladder (between 20 and 120 min), which indicates renal excretion of radiometabolites and/or parent tracer (Figure 5F). The right thigh was used to represent muscular uptake of [^18^F]fluoro-proline. The collected data showed relatively stable muscle activity from 30–60 min to 90–120 min. The tissues/organs with the lowest uptake of [^18^F]fluoro-proline were the spleen, lungs and the brain, with slightly higher activity in the stomach and colon.

**TABLE 1:** Time course of [^18^F]fluoroproline distribution using PET/CT in different organs of normal mice.

	Tracer activity (MBq/L) post radiopharmaceutical injection			
	
Organs	0–20 min	21–40 min	41–60 min	90–120 min
Brain	37 ± 4−	27 ± 4	13 ± 1	11 ± 1
Lung	14 ± 3	11 ± 1	9 ± 1	9 ± 1
Spleen	14 ± 3	9 ± 1	8 ± 1	7 ± 1
Colon	41 ± 4	56 ± 4	43 ± 15	39 ± 6
Stomach	43 ± 5	40 ± 5	33 ± 4	29 ± 3
Pancreas	42 ± 7	39 ± 4	30 ± 2	26 ± 4
Thigh	85 ± 6	74 ± 5	67 ± 5	57 ± 8
Spine	145 ± 27	151 ± 18	124 ± 13	121 ± 11
Heart	302 ± 37	142 ± 24	70 ± 9	43 ± 4
Liver	307 ± 26	158 ± 12	109 ± 13	104 ± 9
Kidney	1007 ± 97	1212 ± 147	747 ± 113	659 ± 47
Bladder	677 ± 32	1114 ± 160	1018 ± 184	1022 ± 171

An initial concern was that [^18^F]fluoro-proline uptake in the spinal region on PET/CT could represent free [^18^F]fluoride, since free [^18^F]fluoride is a well-known bone imaging agent. To address this concern, PET/CT images were repeated in the same normal mouse after injecting the bone-seeking agent, [^18^F]fluoride. A side-by-side comparison of the 2 PET/CT images (Figures 5G,H) showed a clear distinction between [^18^F]fluoro-proline and [^18^F]fluoride images. [^18^F]fluoride uptake was predominantly within the osseous structures of the spinal vertebral bodies (Figure 5J). Moreover, there was no hepatic uptake on [^18^F]fluoride PET/CT imaging (Figure 5H). [^18^F]fluoro-proline uptake was mainly localized to the vertebral fibro-components with the best visualization on the sagittal views (Figure 5I), though the presence of [^18^F]fluoride could not be excluded in the animals that had [^18^F]fluoro-proline PET/CT imaging.

### Detection of Early-Stage Liver Fibrosis in Mice With Acute Steatohepatitis Using Static [^18^F]fluoro-Proline PET/CT Imaging

**FIGURE 6:**
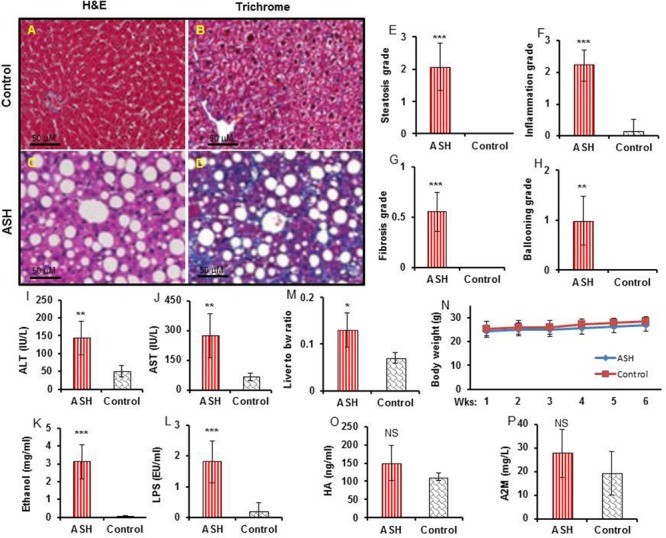
Histopathology, blood biochemistry, and body weight changes in acute steatohepatitis mouse model compared with control mice. **(A,C)** Control and acute steatohepatitis (ASH) mouse liver stained with H&E. **(B,D)**, Control and ASH mouse liver stained with Mason’s trichrome stain. **(E–H)** Summary regarding grades of steatosis **(E)**, inflammation **(F)**, fibrosis **(G)**, and ballooning **(H)** in livers of ASH mice (red straight line, *n* = 7) vs. control mice (gray slanted line, *n* = 7). **(I,J)** Two plasma hepatic enzymes, alanine aminotransferase (ALT) and aspartate aminotransferase (AST), were measured in the serum of mice with ASH (red straight line, *n* = 7) vs. control mice (gray slanted line, *n* = 7). **(K,L)** Ethanol and LPS plasma levels in mice with ASH (red straight line, *n* = 7) vs. control mice (gray slanted line, *n* = 7). **(M)** Ratio of liver weights to body weights. **(N)** Body weight changes during the ethanol feeding periods. **(O,P)** hyaluronic acid (HA) and alpha-2 macroglobulin (A2M) concentrations in mice with ASH (red straight line, *n* = 7) vs. control mice (gray slanted line, *n* = 7). *p < 0.05, **p < 0.01 and ***p < 0.001 vs. corresponding controls. NS, no significant difference between mice with ASH and the control mice.

[^18^F]fluoro-proline PET/CT *in vivo* imaging was applied to evaluate early-stage liver fibrosis in acute steatohepatitis mice. The characteristics of early-stage liver fibrosis were confirmed by histopathology, blood chemistry, and clinical factors. Histopathology of control livers showed no lipid deposits, no inflammatory infiltrates, and no fibrosis (Figures 6A,B). In contrast, significant lipid deposits, moderate inflammation, and minimal fibrosis consistent with early-stage liver fibrosis was seen in acute steatohepatitis mice (Figures 6C,D). The inflammatory markers of lipid deposits (*p* < 0.001, Figure 6E), inflammatory infiltrates (*p* < 0.001, Figure 6F), collagen accumulation (*p* < 0.001, Figure 6G), and ballooning formation (*p* < 0.001, Figure 6H) were also significantly increased in acute steatohepatitis mice compared to control mice. Plasma ALT (144 ± 48 IU/L) and AST (275 ± 111 IU/L) in acute steatohepatitis mice were significantly increased compared with ALT (50 ± 15 IU/L) and AST (65 ± 19 IU/L) in control mice (*p* < 0.01, Figures 6I,J). As expected, plasma ethanol levels in acute steatohepatitis mice were significantly higher than that of controls (3 ± 1 mg/mL vs. 0.1 ± 0.02 mg/mL, *p* < 0.001, Figure 6K). Similarly, plasma LPS concentrations in acute steatohepatitis mice were significantly higher than controls (2 ± 0.7 EU/mL vs. 0.2 ± 0.3 EU/mL, *p* < 0.001, Figure 6L). There were no significant differences between plasma levels of fibrotic biomarkers, HA and A2M, between acute steatohepatitis mice and controls (Figures 6O,P). Liver to body weight ratio was higher in acute steatohepatitis mice than its controls (Figure 6M). There was no difference in body weights at baseline (acute steatohepatitis mice: 24 ± 2g; controls: 25 ± 2g; *p* = NS; Figure 6N) or at the end of the study period (acute steatohepatitis mice: 27 ± 2g; controls: 28 ± 2g; *p* = NS; Figure 6N).

**FIGURE 7:**
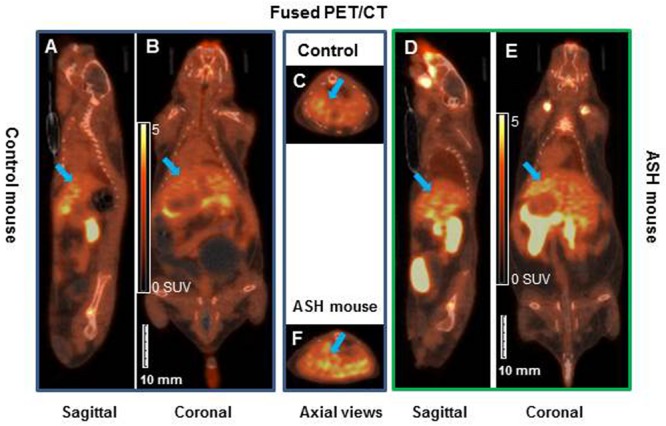
Static [^18^F]fluoro-proline fused PET/CT imaging in acute steatohepatitis mouse model compared with control mice. **(A–C)** Sagittal, coronal and transaxial views of [^18^F]fluoro-proline positron emission tomography/computed tomography (CT) (PET/CT) imaging in control mouse. **(D–F)** Sagittal, coronal and axial views of PET/CT imaging in mouse with acute steatohepatitis. Images were performed 60 min after [^18^F]fluoro-proline was administrated via the tail vein and total imaging length was acquired for 30 min. Green arrow indicates livers of the animals. The representative mouse body weights were 32 g of control mouse and 32 g of ASH mouse and the injected doses were 10 MBq, respectively, of [^18^F]fluoro-proline at the time of imaging experiments.

[^18^F]fluoro-proline PET/CT static imaging was performed 60 min after [^18^F]fluoro-proline was administrated via the tail vein and images were acquired for 30 min. The doses recorded before each experiment prior to injection were 8.0 ± 0.8 MBq in the acute steatohepatitis mice and 8.0 ± 1.0 MBq in the controls. Multiple representative views of static [^18^F]fluoro-proline PET/CT imaging are depicted in Figure 7 including fused sagittal PET/CT (A, D), coronal PET/CT (B, E), and axial PET/CT (C, F) in the acute steatohepatitis mice and controls.

**FIGURE 8:**
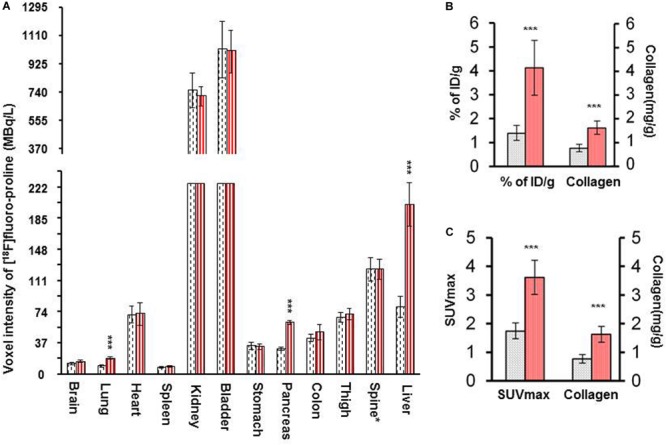
[^18^F]fluoro-proline PET imaging quantification of liver [^18^F]fluoro-proline uptake in acute steatohepatitis mice vs. control mice. **(A)** Voxel intensity of [^18^F]fluoro-proline activity in lung, pancreas, and liver and other 9 organs of acute steatohepatitis (ASH) mice (red column, *n* = 4) and control mice (gray column, *n* = 4). **(B)** Percentage of injected doses per gram body weight of [^18^F]fluoro-proline in livers of ASH mice model and control mice. **(C)** [^18^F]fluoro-proline activity in SUVmax in the livers of ASH mouse model and its controls. **(B,C)** Collagen type 1 concentrations in livers of these two groups (*n* = 7). ***p < 0.001 vs. corresponding controls.

There was a significant increase in [^18^F]fluoro-proline activity in the liver of acute steatohepatitis mice (201 ± 26 MBq/L) compared with control mice (80 ± 13 MBq/L, *p* < 0.001, Figure 8A). There was also increased uptake in the lungs of acute steatohepatitis mice (19 ± 1 KBq/mL) compared with control mice (9 ± 1 MBq/L, *p* < 0.001), and the pancreas of acute steatohepatitis mice (61 ± 2 MBq/L) compared with control mice (30 ± 2 MBq/L, *p* < 0.001) (Figure 8A). There was no statistically significant difference in [^18^F]fluoro-proline activity in the other 9 organs (brain, heart, spleen, kidneys, bladder, stomach, colon, thigh and spinal tube fibrotic components). Blood pool activity levels (based on measurements of left ventricular tracer activity) were similar between acute steatohepatitis mice and controls.

Quantification of liver [^18^F]fluoro-proline activities in terms of % of ID/g (4.0 ± 1.0 in acute steatohepatitis mice vs. 1.0 ± 0.3 in controls, *p* < 0.001) (Figure 8B); SUVmax (4.0 ± 0.6 in acute steatohepatitis mice vs. 2.0 ± 0.3 in controls, *p* < 0.001) (Figure 8C); all indicated a significant increase of [^18^F]fluoro-proline uptake in acute steatohepatitis mice compared with control mice. Furthermore, liver collagen levels (expressed as collagen concentration in mg/mL) were significantly increased in acute steatohepatitis mice (2.0 ± 0.2 mg/g liver tissue) compared with control mice (0.8 ± 0.1, *p* < 0.001). There was an excellent correlation between liver collagen concentrations of ASH mice with both [^18^F]fluoro-proline quantification parameters: (*r* = 0.93 for% ID/g body weight vs. collagen; *p* < 0.001, and *r* = 0.88 for SUVmax vs. collagen; *p* < 0.001) (Figures 8B,C).

## Discussion

The purpose of this study was to evaluate [^18^F]fluoro-proline as a PET tracer for assessment of HSC activation by quantifying proline uptake in early-stage liver fibrosis in mice. HSCs are the primary cells in the liver that produce collagen and were investigated at the cellular level *in vitro* in liver tissue and via non-invasive molecular imaging *in vivo* in the acute steatohepatitis model. Activation of HSC and integration of proline in collagen synthesis (collagenogenesis) are the hallmarks of progressive liver fibrosis from early reversible liver fibrosis to late irreversible cirrhosis, which is characterized by increased collagen deposition (Friedman et al., 1985; Maher and McGuire, 1990). Fibrosis can, therefore, be considered collagen accumulation over time. Proline is the principle amino acid in collagen synthesis, where it is enriched nearly 5-fold more than other proteins (Morgan and Rubenstein, 2013). Proline and hydroxyproline comprise ∼23% of the amino acid content of the collagen molecule (Grant and Prockop, 1972; Krane, 2008). To form hydroxyproline, proline hydroxylation occurs post-translationally and is carried out by the enzyme, prolyl hydroxylase. 99.8% of the body’s stores of hydroxyproline are found in collagen, rendering assays of this amino acid useful as a marker for the total amount of collagen.

In the current study, we applied radioisotope-labeled proline ([^18^F]fluoro-proline) to target collagenogenesis in the liver using micro-PET/CT. We found (1) significantly higher [^18^F]fluoro-proline uptake in the livers of early-stage liver fibrosis compared with its normal controls, and (2) significantly higher intracellular α1(1) procollagen mRNA expression in the HSCs of acute steatohepatitis mice with early-stage liver fibrosis compared with those of control mice. The findings from our *in vitro* [^3^H]proline experiments and *in vivo* [^18^F]fluoro-proline PET imaging studies suggest that [^18^F]fluoro-proline PET/CT may be a useful tool to assess collagen synthesis activation of HSCs, which are the primary cells involved in the process of collagen formation and fibrosis (Carey and Carey, 2010; Skovgaard et al., 2011). Increased HSC activation with associated increased collagen gene expression and collagen protein synthesis was significant in the stage of acute steatohepatitis in LPS-treated mice fed with the alcohol diet compared with control mice that were not administered LPS.

Alcoholic hepatitis is a form of acute-on-chronic liver injury frequently mediated by gut derived LPS (Affò et al., 2014; Odena et al., 2016). To establish experimental acute alcoholic steatohepatitis, LPS was administered by intravenous or intraperitoneal injection into animals fed with alcohol diets, including rats and mice (Batey et al., 1998; Lowe et al., 2017; Perea et al., 2017). Unlike patients with alcoholic hepatitis, mice or rats have minimal inflammation associated with being chronically fed an alcohol diet, especially in short term feeding periods less than 3 months duration. Our current experiment model aimed to mimic acute alcoholic steatohepatitis, thus we used LPS injection to induce acute hepatitis. There was 2–3-fold higher [^18^F]fluoro-proline uptake in the livers of mice with acute steatohepatitis than that of control mice. Based on these findings, it is conceivable that [^18^F]fluoro-proline PET/CT could identify early liver damage, which would permit tailoring medical therapies to individual needs, and improve patient outcome. In addition, this molecular imaging strategy would allow for the non-invasive monitoring of disease progression over time, without exposing the patient to the undue risk of repeated liver biopsies. While liver biopsy is the current “gold standard” for making the diagnosis and characterizing the extent of hepatocellular damage, it is generally accepted that sampling error could underestimate the magnitude of liver pathology (Carey and Carey, 2010; Morgan et al., 2013). Moreover, given the invasive nature of the biopsy procedure and the associated morbidity and mortality, monitoring disease progression with repeat biopsies is inadvisable.

Because of the absence of [^18^F]fluoro-proline and its radiometabolites in bile and intestines, [^18^F]fluoro-proline is an ideal liver imaging agent that does not have confounding artifacts related to biliary excretion, gallbladder accumulation, and bowel excretion. These findings are in line with prior biodistribution studies in mice and humans (Wester et al., 1999; Borner et al., 2001; Skovgaard et al., 2011). [^3^H]proline uptake was seen predominantly in HSCs isolated from acute steatohepatitis livers, as opposed to liver inflammatory cells, Kupffer cells, and liver parenchyma cells, hepatocytes. Increased expressions of collagen and proline transporter were observed in both rat and mouse steatohepatitis models compared with that of controls, but no significant difference was seen in their expression between rat and mouse models. These findings suggest that the mouse model is the preferred model to measure HSC activation using non-invasive imaging, because of a lower radiotracer dose and experimental costs (such as animal housing and food consumption) compared with using rats. A greater increase in collagen synthesis from baseline levels was found when the liver tissues of mice with acute steatohepatitis were incubated with proline compared with those of the control mice, but less [^3^H]proline was taken up as the concentration of unlabeled fluoro-proline increased, suggesting that the mechanism of proline uptake in the liver is saturable. It is interesting to note that proline transporter inhibitors could bring proline-increased collagen synthesis back to baseline levels, with an associated decreased [^3^H]proline uptake. These findings suggest increased collagen synthesis might be involved in HSC activation mainly via elevated proline transporter expression. Alternatively, it is possible that the lack of proline prevents collagen synthesis by otherwise activated HSC. Based on this finding, we hypothesize that quantification of [^18^F]fluoro-proline activity in the liver could be used to represent activated HSC collagen synthesis via elevated proline transporter expression, which is an area for future studies. The specificity of [^3^H]proline uptake into other intrahepatic inflammatory cells including neutrophils and T cells is another area of future research. Similarly, [^18^F]proline uptake in the trabecular component of the skeleton in this study supports collagen turnover as a source of trabecular bone activity rather than contamination from free, unbound ^18^F. Additional imaging studies performed in the same animals with [^18^F]fluoride showed [^18^F]fluoride was localized primarily within the bony structures rather than trabecular component of the skeleton. However, this study could not exclude [^18^F]floride contamination in the radiotracer, [^18^F]fluoro-proline.

Currently available clinical imaging techniques are unable to detect the earliest hepatic fibrotic changes. Structural imaging, such as CT or MRI, can identify macrostructural changes of liver nodularity in later “irreversible” stages of fibrosis. At the present time, there is a gap between serum fibrotic biomarkers, non-invasive imaging parameters and clinical indications for progressive liver fibrosis. Thus, one of the important goals of molecular imaging probes and techniques is to identify early “reversible” changes or signals in the disease process, prospectively, before the transition to replacement fibrosis and remodeling of the liver occurs, which may result in preserved hepatic function and thereby improve overall patient survival. Functional techniques such as diffusion and elastography are other promising techniques for diagnosing moderate-to-severe fibrotic changes; however, they are less reliable for diagnosing mild (early stages) fibrosis (Bonekamp et al., 2009; Denzer and Luth, 2009; Thiele et al., 2017). Similarly, despite ongoing research efforts with ultrasound elastography, which is operator dependent, reliable results are only found in late stages of fibrosis and concerns exist regarding data reproducibility. While MR elastography has improved reproducibility, it remains less specific at early stages of fibrosis as many other pathologic processes can affect liver stiffness, such as transient hepatitis. Furthermore, although MR elastography is an emerging field under active investigation, it remains widely unavailable and requires expensive specialized coils. An MR molecular imaging technique under active investigation is a gadolinium-based molecular probe for type 1 collagen, which has shown some success in the early diagnosis and staging of hepatic fibrosis in a CCl_4_-treated mouse model (Fuchs et al., 2013). However, positron-emitting radiopharmaceuticals offer high contrast resolution at the molecular level, and thereby identify subtle changes that occur early in the disease process. Molecular imaging with PET is more sensitive than MRI by several orders of magnitude and requires less scanning time. PET provides the ability to quantify the signal through a variety of methods, which could be helpful in the early staging of liver fibrosis. Late-stage liver fibrosis can be readily staged by many other imaging modalities including MRI, CT, and ultrasonography. Our primary findings of increase in [^18^F]fluoro-proline activity in the liver of experimental alcoholic steatohepatitis will encourage us to continue this line study toward patients with alcoholic steatohepatitis. Our future [^18^F]fluoro-proline PET imaging studies will continue in experimental alcoholic liver disease models and patients with steatosis, early stage liver fibrosis, and late stage liver fibrosis.

Incidental findings of increased [^18^F]fluoro-proline activity in the lungs and pancreas in alcohol diet-fed mice administrated with LPS compared with those not administered LPS might be consistent with other non-imaging findings regarding activation of collagen producing cells (Vonlaufen et al., 2007; Mitchell et al., 2009; Zhang et al., 2013; Sueblinvong et al., 2014). Feeding of Lieber-DeCarli ethanol diets rendered the experimental mouse lungs susceptible to fibrosis by activated pulmonary collagen-producing cells following bleomycin-induced acute lung inflammation and lung transplantation (Mitchell et al., 2009; Sueblinvong et al., 2014). Lieber-DeCarli alcohol diet intake perpetuated pancreatic injury by inhibiting apoptosis and promoting activation of pancreatic stellate cells (PSCs) in rats challenged with single dose of LPS (Zhang et al., 2013). Repeated LPS injections caused pancreatic fibrosis in alcohol-fed rats, but not in rats fed the control diet. In further *in vitro* study by this group showed that PSCs were activated by LPS and that Alcohol + LPS exerted a synergistic effect on PSC activation. Further [^18^F]fluoro-proline imaging studies are necessary to evaluate pulmonary collagen-producing cell and PSC activation via the stimulation of fibrogenic mediators including transforming growth factor and LPS in our experimental models. The signal difference in pancreas may be explained by errors in drawing ROIs due to overlap with the liver. The radiotracer uptake in the lungs is less likely affected by overlying liver because of the clearly defined diaphragm between the two organs.

## Conclusion

[^18^F]fluoro-proline PET/CT is a useful, non-invasive imaging technique for quantifying collagenogenesis in terms of HSC activation and proline integration into collagen synthesis during the early stages of alcoholic liver fibrosis which cannot be diagnosed using the current MRI and CT techniques. [^18^F]fluoro-proline localizes in the liver and detects proline uptake in early-stage liver fibrosis with a signal intensity that is sufficiently high to allow non-invasive imaging with micro-PET/CT. Thus, [^18^F]fluoro-proline could serve as a PET imaging biomarker for liver fibrosis detection in diseases, such as steatohepatitis.

## Data Availability Statement

The raw data supporting the conclusions of this article will be made available by the authors, without undue reservation, to any qualified researcher.

## Ethics Statement

The animal study was approved by the committee on the Ethics of Animal Experiments of University of Maryland School of Medicine.

## Author Contributions

QC, PI of the project, oversaw project, and wrote the manuscript. XL conducted small animal PET/CT imaging. BA and MP collaborators from JHU, performed synthesis of [^18^F]proline, provided guidance regarding imaging studies, and revised the manuscript. JH collaborator, provided guidance regarding imaging studies, and revised the manuscript. MS oversaw aspects regarding nuclear physics, microPET/CT operation, radiation safety, and revised the manuscript. LP, BR, and ZY collaborators who worked with animal models and lab studies and revised the manuscript. BS and MM provided research assistance, and wrote the data analysis portion of the manuscript. VD and QC’s mentor in the K08 NIH grant, provided guidance regarding the project and its progression and helped revise the manuscript.

## Conflict of Interest

The authors declare that the research was conducted in the absence of any commercial or financial relationships that could be construed as a potential conflict of interest.

## Abbreviations

ALT: alanine aminotransferase
A2M: alpha-2-macroglobulin
AST: aspartate amino transferase
CPM: counts per minute
[18F]fluoro-proline: cis-4-[18F]fluoro-L-proline
HSC: hepatic stellate cells
HA: hyaluronic acid
H&E: hematoxylin and eosin
ID: injected dose
LPS: lipopolysaccharide
MBq/L: megabecquerel per liter
PET/CT: positron emission tomography/computed tomography
qRT-PCR: quantitative real-time polymerase chain reaction
SD: Sprague-Dawley
RP-HPLC: reversed-phasehigh-performance liquid chromatography.
